# Preclinical evaluation of the polycaprolactone-polyethylene glycol electrospun nanofibers containing egg-yolk oil for acceleration of full thickness burns healing

**DOI:** 10.1038/s41598-023-28065-6

**Published:** 2023-01-17

**Authors:** Vida Shadman-Manesh, Adeleh Gholipour-Kanani, Najmeh Najmoddin, Shahram Rabbani

**Affiliations:** 1 Department of Biomedical Engineering, Science and Research Branch, Islamic Azad University, Tehran, Iran; 2 Department of Textile Engineering, Science and Research Branch, Islamic Azad University, Tehran, 1477893855 Iran; 3 Research Center of Advanced Technologies in Cardiovascular Medicine, Cardiovascular Diseases Research Institute, Tehran University of Medical Science, Tehran, Iran

**Keywords:** Preclinical research, Nanoscale materials, Biomaterials

## Abstract

Considering the great potential of egg yolk oil (EYO) in management of burn wounds and superb biological properties of polycaprolactone (PCL) and polyethylene glycol (PEG), hereby, a PCL-PEG-EYO scaffold was developed by electrospinning method for burn healing. The physico-chemical characterizations were performed using SEM, FTIR and contact angle tests. The biological properties of the fabricated scaffolds were evaluated by antibacterial test, in vitro cell culturing, MTT assay and in vivo experiments. The SEM images of PCL-PEG-EYO nanofibers demonstrated a uniform bead-free morphology with 191 ± 61 nm diameter. The fabricated scaffold revealed hydrophilicity with the water contact angel of 77°. No cytotoxicity was observed up to 7 days after cell culturing onto the PCL-PEG-EYO nanofibrous surface. The presence of EYO in the PCL-PEG-EYO scaffold meaningfully improved the cell viability, proliferation and attachment compared to PCL-PEG scaffold. Moreover, the PCL-PEG-EYO scaffolds demonstrated antibacterial activity against *Staphylococcus aureus* and *Pseudomonas aeruginosa* bacteria strain. Finally, a statistically significant enhancement in wound closure, re-epithelialization, angiogenesis and collagen synthesis was observed at the end of 21-day treatment period using PCL-PEG-EYO nanofibrous scaffold. Overall, the PCL-PEG-EYO nanofibrous scaffolds demonstrated a great potential in management of full thickness burn wounds in vivo.

## Introduction

Burn wounds are among the most frequently occurring traumas, accounting for more than 256,000 deaths worldwide^1^. Depending on the depth and severity of penetration through the skin's surface, burns are sub-classified as first, second or third degree. In the third-degree burns (full-thickness) as the most serious type, the injury will exceed beyond the dermis layer and reach into the hypodermis^2^. The wound healing process consists of several critical steps comprising hemostasis, inflammation, proliferation and wound remodeling^3,4^. The mechanisms underlying the mentioned steps are as follows: (i) growth factors and inflammatory mediators; (ii) cell–cell and cell–extracellular matrix interactions that govern cell migration, proliferation, and differentiation; (iii) events involved with epithelialization, fibroplasia and angiogenesis; (iv) wound contraction; and (v) remodeling^5^.

One of the most important considerations in burn wound care is protecting it from bacterial infection^6^. It has been revealed that infection of wounded area will cause a delay of healing process, induction of scarring and if not properly treated will end up with development of bacteremia, septic shock and multiple organ dysfunction syndrome^7^.

Exploiting biomedical materials such as fibrous structure of biopolymer blends explore superb characteristics such as a large specific surface, a 3D-network structure and a high porosity which are amazing for tissue engineering. The electrospun nanofibrous structures are among the golden standards of burn wound care which could recover physical properties of the skin. These structures specially when they are containing drug, could accelerate cell repairing processes and play the role of a protective shield against induction of infection^8,9^. Moreover, an optimal dressing can absorb the extravagated interstitial fluid during the repair^10^. Poly(ɛ-caprolactone) (PCL) and poly(ethylene glycol) (PEG) are two non-cytotoxic, biocompatible and biodegradable polymers which possess excellent permeability^11,12^. Combining biological and mechanical properties of PCL with drug delivery capacity of PEG as a biocompatible lubricant agent were studied in our previous report in which PCL: PEG: Aloe-Vera nanofibers had been fabricated and evaluated for bone repairing application^13^. According to the other reports, the properties of PCL and PEG have turned them in to frequently used raw materials in development of several burn wound care dressings which have succeeded in receiving FDA-approval^14,15^.

Egg yolk oil (EYO), achieved by purifying cooked egg yolks, comprises a range of essential nutrients such as saturated and unsaturated fatty acids, fat miscible vitamins, phospholipids and cholesterol^16^. It has long been applied as a remedy for burn wounds in Chinese traditional medicine^17^. From the literature, the immunoglobulin in egg yolk can destroy pathogens and inhibit the growth of bacteria^18^. Today, EYO is commonly applied in treatment of all types of burn wounds. For instance, Rastegar et al.^19^ have successfully utilized EYO in the management of the third degree burns created in rats and showed a statistically significant enhancement in re-epithelialization process without any signs of scarring compared to the silver sulfadiazine as a control group. Moreover, Zhao and co-workers^20^ demonstrated that EYO could induce activation of the fibrinogen through the intrinsic coagulation pathway and produce a powerful hemostatic activity. Additionally, presence of negatively charged lecithin in the EYO would induce electrostatic attraction with the positively charged polymers from one side and repulsion with negatively charged emulsified oil droplets from other side which consequently stabilizes the emulsions and prevents coalescence and separation of oil in polymer solution^21^. This could be the reason for popularly employed of lecithin in emulsification and mixing of hydrophobic drugs with hydrophilic polymers such as chitosan. Yenilmez et al.^22^ stated that a mixture of EYO with chitosan gels could effectively accelerate local healing of dermal burns, demonstrating excellent restorative activities. Rodil et al.^23^ demonstrated the accelerating surface adhesiveness and cellular growth of 3T3 fibroblast cells using EYO.

To the best of our knowledge, fabrication of a fibrous dressing composed of PCL and PEG with EYO through electrospinning process has not been reported yet. Morphological, Physical, antibacterial tests as well as cytotoxicity assay were performed to characterize the fabricated construction. Moreover, the efficiency of such engineered dressing in third degree burns healing and preventing wound infection were investigated using in vivo experiments on rat model.

## Materials and methods

### Materials

The PCL (*M*_*w*_ = 80,000 g/mol) and PEG (*M*_*w*_ = 8000 g/mol) were obtained from Sigma-Aldrich (St. Louis, MO, USA). EYO was purchased from Taqdis Company (Tehran, Iran) and dichloromethane (DCM) and dimethyl formamide (DMF) were obtained from Merck (Darmstadt, Germany). Moreover, Phosphate Buffer Saline (PBS), fetal bovine serum (FBS), Dulbecco's Modified Eagle Medium/ Nutrient Mixture F-12 (DMEM/F12) cell culture medium and antibiotics were acquired from Invitrogen (Carlsbad, CA, USA). The 3-(4,5-dimethylthiazol-2-yl)-5-(3-carboxymethoxyphenyl)-2-(4-sulfophenyl)-2H-tetrazoli solution (MTT) was purchased from Promega (Fitchburg, WI, USA). All reagents were of analytical grade and were used as received without further purification.

### Preparation of PCL-PEG and PCL-PEG-EYO nanofibrous scaffolds

PCL:PEG solutions in 1:1 and 2:1 mass ratios were prepared by dissolving 0.25 g (for 1:1 ratio) or 0.5 g (for 2:1 ratio) of PCL in 5 mL of DCM: DMF (9:1) and stirring for 1 h at 40 °C. Afterwards, 0.25 g PEG was added to the abovementioned solutions and stirred for another 1 h to achieve homogenous and clear solutions. For preparation of PCL-PEG-EYO, the same process was performed and finally 0.05 g EYO was added to the mixture and stirred for 1 h.

Following the preparation of solutions, a classical electrospinning approach (FNM Duos Electroris, HV35P OV, Fanavaran Nanomeghyas Co., Iran) was applied utilizing a positive high voltage source (10, 15 and 20 kV), a 3 mL syringe with a 20 G needle and a stainless-steel drum as a collector covered by a aluminum foil (needle tip to collector distance of 10, 15 and 20 cm) at infusion speed of 1 mL/h, temperature of 23 °C, and 40% humidity. Schematic representation of the experimental procedure was demonstrated in Fig. S1.

### Characterization of the nanofibrous scaffolds

#### Morphological and physicochemical characterizations

Scanning electron microscopy (SEM) was used for evaluation of the morphology of the nanofibers to determine the optimum condition of electrospinning process. In brief, the nanofibrous scaffolds were initially dried and the samples were then mounted on aluminum stubs, followed by sputter-coating with gold, and imaging using a JEOL SEM device (JSM5300, JEOL Ltd., Tokyo, Japan).

FT-IR spectroscopy was performed to study the chemical bonds of the scaffolds and define probable interactions between components in the blends. In brief, specimens were combined with potassium bromide powder and pressed to create a round tablet. The FT-IR spectra of the specimens were then acquired by a FT-IR spectrophotometer (Bruker, Germany) and analyzed in the range of 400–4000 cm^−1^.

The hydrophilicity of the fabricated webs was assessed by measuring the contact angle of distilled water drop on the web, using Kruss drop shape analyzer (DSA 100). The webs were cut into 100 mm^2^ and fixed into the custom made sample holder. The shape of a single drop of distilled water (volume∼2 μL) on the web surfaces was recorded using the camera attached to the drop shape analyzer after 3 s. The contact angle was measured by the sessile drop approximation of the inbuilt software of the instrument.

#### In vitro cell culture studies

To evaluate cell viability using MTT assay, as prepared PCL-PEG and PCL-PEG-EYO nanofibrous scaffolds were sterilized by 70% ethanol for 1 h. Following evaporation of the ethanol, specimens were washed three times in PBS to remove any remaining ethanol. Scaffolds were then placed in DMEM/F12 medium and incubated for 4 days at 37 °C to reach equilibrium with medium prior to initiation of cell seeding.

L929 cells were collected from flasks using 0.05% trypsin upon reaching 80% confluency and seeded at a concentration of 10^4^ cells per scaffold in 12-well plates and kept in DMEM/F12 medium supplemented with 10% fetal bovine serum (FBS) and 1% penicillin/streptomycin solution. The specimens were incubated for 1, 3 and 7 days under the humid atmosphere containing 5% of CO_2_ at 37 °C. On predetermined time points, the previous media were removed and replaced with 200 µL MTT solution (2 mg/mL) together with 400 µL DMEM/F12 medium and incubation continued for 4 h at 37 °C. Then, the whole medium was replaced by 600 µL dimethyl sulfoxide (DMSO) to dissolve formed purple formazan crystals. Finally, the absorbance of the solution was read at 570 nm using an ELISA reader (BioTek, Winooski, VT, USA). For each specimen, experiments were done in triplicate.

The cell morphology and distribution on the scaffolds were observed by SEM. After each time point (1, 3 and 7 days), the samples were washed with PBS for 30 s, and then 3.5% glutaraldehyde was used for cell fixation. Then, the samples were placed in the refrigerator for 2 h, the fixator was removed, and the specimens were washed with deionized water and 50%, 60%, 70%, 80%, and 96% alcohol, respectively and finally air-dried.

#### Antibacterial test

Initial data on antibacterial activity of the PCL-PEG and PCL-PEG-EYO nanofibrous scaffolds was gathered through the performance of the agar well diffusion test. In this context, *Staphylococcus aureus* (ATCC 25923) and *Pseudomonas* *aeruginosa* (ATCC 90271) bacteria were applied as gram positive and negative strains for evaluation of the antibacterial activities of the scaffolds. In brief, each scaffold was carefully punched to achieve an 8 mm in diameter discs and sterilized by immersing in 70% ethanol for 45 min and subsequently exposing to 1 h UV irradiation. Suspensions from *P. aeruginosa* and *S. aureus* at a final concentration of 10^5^ CFU/mL were prepared using distilled salinized water (0.9% NaCl) and plated on 3-(N-Morpholino) Propane-Sulfonic Acid (MOPS) medium (Liofilchem, Italy) and Baird–Parker agar (Liofilchem, Italy) respectively. Sterilized samples were then placed on the petri dishes and incubation continued for 24 h at 44 °C and 36 °C for *P. aeruginosa* and *S. aureus*, respectively. Finally, diameters of the inhibition zones were measured in relation to the diameter of the petri dish (6 cm). Each experiment was performed in triplicate.

The antimicrobial activity of EYO was evaluated by determining the minimum inhibitory concentration (MIC). For this purpose, *Staphylococcus aureus* (ATCC 25923) and *Pseudomonas* *aeruginosa* (ATCC 90271) were transported to Mueller Hinton Broth medium and put in an incubator at 37 °C for 3 h to obtain 0.5 McFarland. Regards, 1 mg/mL of EYO in phosphate buffer was prepared to form standard stoke solution, and then it was diluted to 0.1 µg/mL which was considered as the reference concentration level of standard. In the next step, 50 μL of each bacterium was added to each tube, to reach the final concentration of 10^6^ CFU/mL. The MIC was determined after incubation for 24 h at 32–35 °C. Each experiment was performed in triplicate.

#### In vivo experiments

All protocols and animal handling procedures were performed in line with the latest guidelines provided by the US National Institutes of Health. The study is reported in accordance with ARRIVE guidelines. Moreover, prior to the initiation of the study, the whole project and the protocols were reviewed and approved by the ethics committee of the Azad University of medical sciences Tehran branch (Code Number: REC.SRB.IAU.IR.025.1400).

Third degree burn wounds were created on Sprague–Dawley rats (n = 12) weighing about 200 ± 20 g while food and water were provided ad libitum. One night prior to the performance of the experiment, hairs on the back of the rats were denuded using 8% Na_2_S aqueous solution ^24^. On the day of the experiment, rats were anesthetized by injecting 50 mg/kg of ketamine and 5 mg/kg xylazine (Sigma Aldrich) intraperitoneally and then, a total of 4 full-thickness burn wounds were created on the dorsal skin of each rat, using a circular brass probe (6 mm in diameter) attached to an iron solder to reach 150 °C and applying for 60 s^25^. All wounds were debrided with pistol and the diameter of the wounded area was about 10 mm after debridement. Each rat received 1 mL physiologic serum subcutaneously for prevention from the occurrence of dehydration. Following the induction of full-thickness burns, wounds were either covered with PCL-PEG and PCL-PEG-EYO nanofibrous dressings or treated with a 0.9% physiologic serum, as a control group. The whole burned areas were fixed using a layer of sterile gauze and an elastic bandage. Wounds were imaged every other day using a digital camera (Canon, Japan) for macroscopic evaluation of the rate of burn wound healing in different groups up to the end of the 21 days post induction of burn injuries. Rats were sacrificed on days 3, 7, 14 and 21 post treatments and the specimens were collected for evaluation of the skin healing process histologically.

#### Histological evaluations

Collected wound specimens were fixed using 4% formaldehyde at 4 °C, dehydrated following introduction to a graded ethanol series, and paraffin-embedded for microtome cuttings and performed different common staining: Masson's trichrome staining followed with CD31 marker and Hematoxylin–Eosin (H&E) staining^26^. Stained slides were then examined under an Olympus light microscope (Olympus, Tokyo, Japan). Masson’s trichrome staining is used to demonstrate the synthesis and deposition of collagen in the healing process; CD31 as an immunohistochemical marker is used for angiogenesis or forming new blood vessels during healing process; and H&E staining demonstrates the general histological architectures of the tissue such as tracking the rate of re-epithelialization^27^.

For evaluation of the healing effects of the nanofibrous scaffolds in detail, different parameters of the wound healing process, including the progress in the extend of the re-epithelialization process, the inflammatory responses (the presence of polymorphonuclear neutrophil (PMNL)), the number of fibroblasts present in the wounded area, the density of the newly formed vessel (angiogenesis) and collagen synthesis and deposition were microscopically examined and scored by an expert pathologist according to the criteria described by Kilik et al.^28^ (Table S1).

#### Statistical analysis

Data were represented as mean of replicates ± standard deviations (SD). One way ANOVA followed by Tukey post *hoc* analysis was applied for statistical comparing of the means in different groups. Whole statistical computations were carried out in SPSS software (v. 16.0; SPSS, Chicago, IL). Differences considered to be statistically significant when the *P* value was lower than 0.05 (*P* < 0.05).

## Results and discussion

### Morphological investigations of nanofibers

Figure S2 represents the morphology of PCL-PEG nanofibers with different mass ratios (PCL: PEG 1:1 and 2:1) which were electrospun at a constant voltage of 20 kV and distance to the collector of 15 cm. As can be seen, the PCL-PEG fibers with 1:1 mass ratio demonstrated bead-free, homogenous and smooth surface morphology with an average diameter of 144 ± 41 nm. Increasing the PCL: PEG mass ratio to 2:1 resulted in a non-uniform and beaded morphology which is obvious at higher magnification. Moreover, increment of the nanofibers diameters up to 410 ± 160 nm has occurred due to the increasing of PCL content in the solution. Since PEG has a low molecular weight, it acts as a lubricating agent between PCL chains, resulting in reduction of the solution viscosity and consequently thinning of the extruded jet. So, nanofibers with lower diameter could be achieved by presence of more PEG in the solution. Contrarily, increasing PCL concentration will accompany by increment of solution viscosity and also tension of the extruded jet, ending in increasing the nanofibers diameter. Hence, the PCL: PEG composition with 1:1 ratio was chosen for further study.

**Figure 1:**
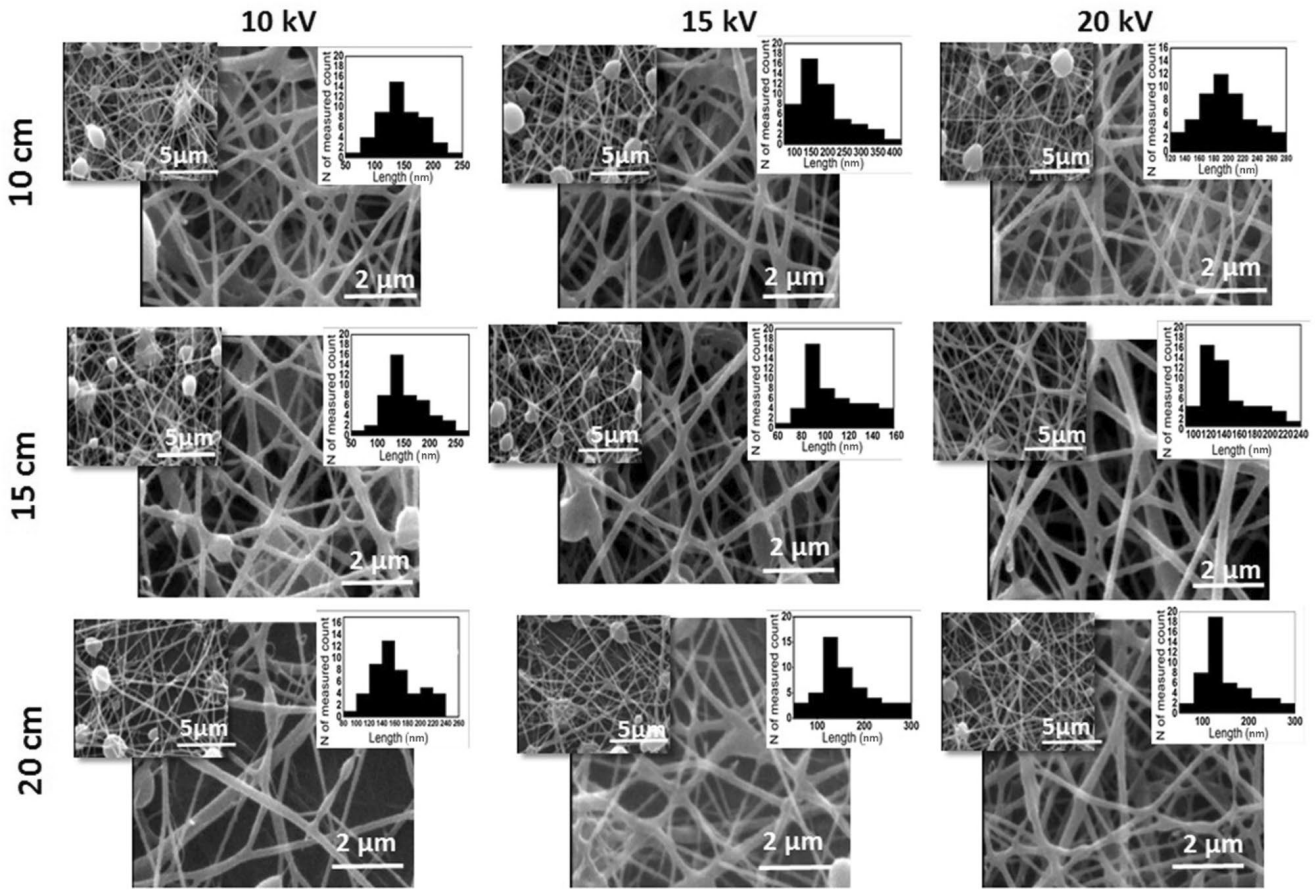
SEM images of PCL-PEG (mass ratio of 1:1) electrospun nanofibers under 10, 15 and 20 kV applied voltage and 10, 15 and 20 cm distance to collector.

**Figure 2:**
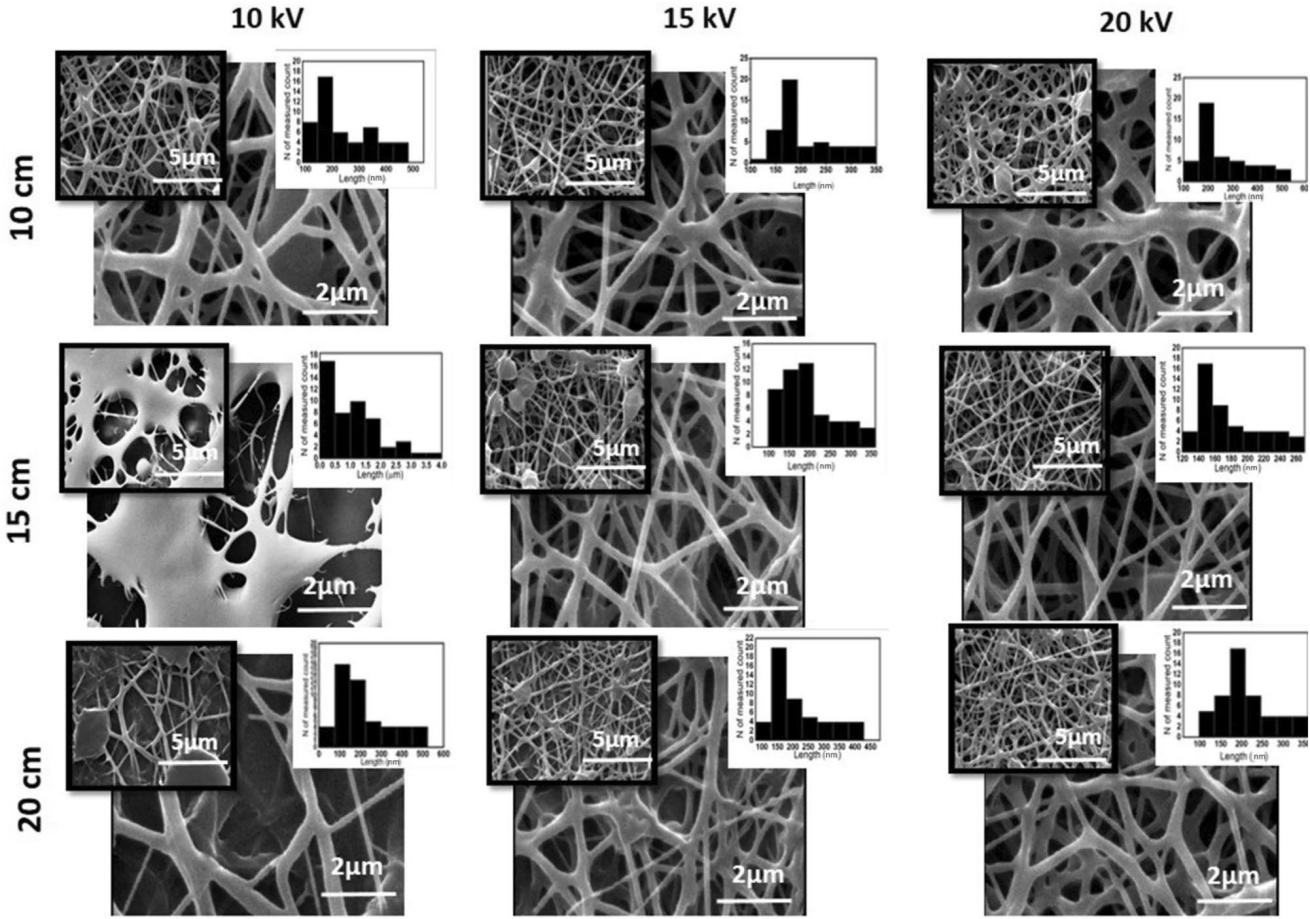
SEM images of PCL-PEG-EYO electrospun nanofibers under different condition of 10, 15 and 20 kV applied voltage and 10, 15 and 20 cm distance to collector.

Figures 1 and 2 represent the morphological characteristics of the fabricated PCL-PEG nanofibers comprising PCL: PEG (1:1) in the absence and presence of EYO, respectively using different electrospinning conditions.

As can be seen in Fig. 1, no significant improvement has occurred on the morphology of nanofibers by increasing the nozzle tip to the collector distance from 10 to 20 cm at a constant voltage of 10 kV and lots of bead could be distinguished in the SEM images of the mats. The diameter of the nanofibers was measured to be 160 ± 60 nm, 158 ± 52 nm and 188 ± 71 nm at 10 cm, 15 cm and 20 cm, respectively. Overall, distance between the nozzle tip and the collector induces two competitive effects on the size of the nanofibers. In the first scenario, the increase in the distance will result in attenuation of the electric field effect on the jet and reduction of tension forces, ending in increment of the nanofibers size. Contrarily, in the second scenario, the increment in the distance will increase the time of jet flight, allowing an extended time for solvent evaporation, result in decreasing of the nanofibers diameter^29–31^.

To evaluate the effect of voltage on morphology and size of nanofibers, electrospinning was performed by setting constant distances and changing voltage between 10 and 20 kV. At a constant distance of 10 cm, increasing the voltage from 10 to 15 kV resulted in a statistically significant increase in nanofibers diameter, reaching to 201 ± 83 nm while enhancing the voltage further to 20 kV effectively reduced the mean size of nanofibers to 94 ± 32 nm. Similar to the effect of the nozzle tip to the collector distance, it has been reported that increasing voltage will end in enhancement of the extrusion jet volume which will result in increasing the nanofibers diameter^13^. On the other hand, increasing the electric field intensity can also result in enhancement of tension forces on polymer, ending in reduction of the nanofibers diameter^32^. It is evident that at a constant distance, the voltage increment resulted in a more uniform and less bead formation which is more pronounce at 20 kV. To conclude, considering bead-free morphology and low fiber diameter, PCL-PEG nanofibers fabricated at 20 kV and 15 cm (144 ± 25 nm diameter) was considered for further evaluation.

Addition of EYO to the solution leads to formation of thicker fibers which can be due to hydrophobic nature of the oil and increasing polymeric chain entanglements. As it depicted in Fig. 2, while non-uniform and weak physically networks were obtained at 10 kV, increasing voltage caused more reliable morphology of fibers. Finally, the solution of PCL:PEG:EYO with mass ratio of 1:1:1 was successfully electrospun under the optimum condition of 20 kV and 15 cm distance and uniform fibers with less bead with an average diameter of 191 ± 61 nm was achieved.

### FT-IR analysis

**Figure 3:**
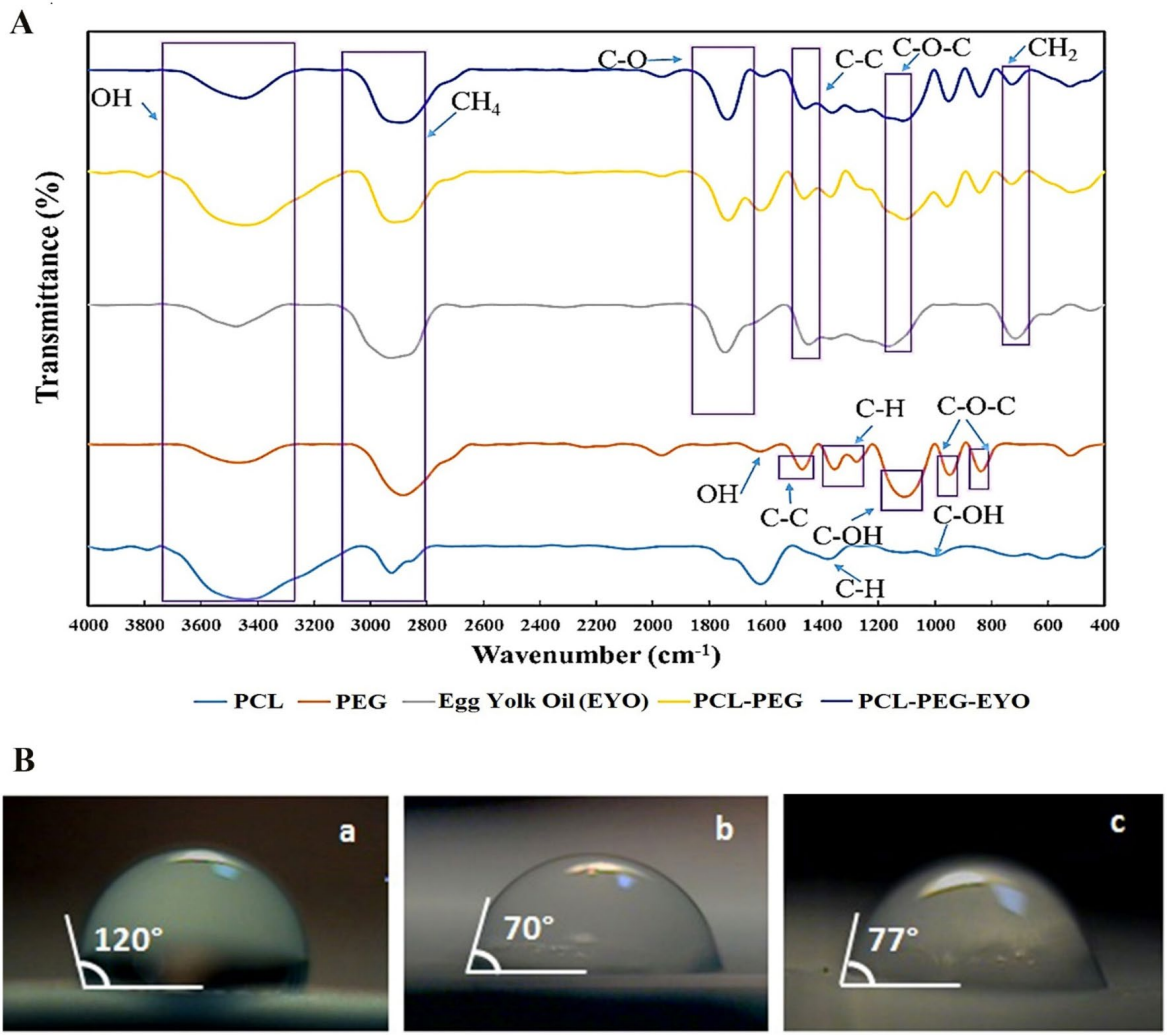
(**A**) FTIR spectra of PCL, PEG and EYO oil followed by the FTIR spectra of PCL-PEG and PCL-PEG-EYO nanofibrous webs; (**B**) Digital images of contact angle of water droplet on (a) PCL, (b) PCL-PEG and (c) PCL-PEG-EYO nanofibrous web.

Figure 3A represents the FT-IR spectra related to PCL, PEG, EYO, PEG-PCL and PCL-PEG-EYO nanofibers. The characteristic peaks of PCL polymer were as follows: –OH stretching band at 3442 cm^−1^, aliphatic ester band at 1732 cm^−1^, and sp^3^ stretching bands of 2858 and 2923 cm^−1^^33^. Regarding PEG, the characteristic peaks comprised of the broad band of hydroxyl group presented between 3400 and 3450 cm^−1^, the C–H aliphatic stretching band at 2889 cm^−1^, and the C = O stretching band located at 1107 cm^−1^^34^.

Moreover, for EYO, these peaks composed of –OH and –NH stretching peak at 3478 cm^−1^, strong –CH symmetrical and asymmetrical stretching bands presented between 2650 and 3000 cm^−1^, C=O stretching peak at 1742 cm^−1^ and –C–O–C peak illustrated at 1151 cm^−1^.

Characteristic peaks related to PCL and PEG in the presence and absence of EYO were jointly observed in the spectra of PCL-PEG and PCL-PEG-EYO composite dressings with a few nanometers shift. This shift can be attributed to the formation of intra- and inter-molecular hydrogen bonds between the -OH and ester groups of PEG and PCL or -OH and -NH groups of EYO with those of PCL and PEG respectively^35^.

### Evaluation of the surface hydrophilicity of the nanofibrous webs

The hydrophilicity of a scaffold is an important determinant of the proper interaction of the scaffold with its biological environment. Many research endeavors suggested that a water contact angel of less than 90 degrees is acceptable for a proper attachment of cells to the surface of the scaffolds^36–38^.

As depicted in Fig. 3B, the water contact angle for PCL is about 120°, proposing the very hydrophobic nature of the PCL polymer-based nanofibers. This can be explained by the presence of numerous hydrophobic groups in the structure of the PCL^13^. Contrarily, addition of the PEG in the structure of the PCL-PEG resulted in reduction of the water contact angel to 70° which can be explained by the hydrophilic nature of the PEG polymer^13^. Regards, a physical crosslinking through the intra-molecular interaction between functional groups of PCL and PEG leads to the integrity of the scaffold in aqueous media. Finally, addition of the EYO in to the PCL-PEG solution resulted in a trivial increase in the water contact angel of the composite, reaching to the value of 77°. Consequently, the PCL-PEG-EYO nanofiber mat demonstrated an acceptable hydrophilicity and presence of EYO did not significantly affect the hydrophilicity of the composite.

### In vitro cell study results

**Figure 4:**
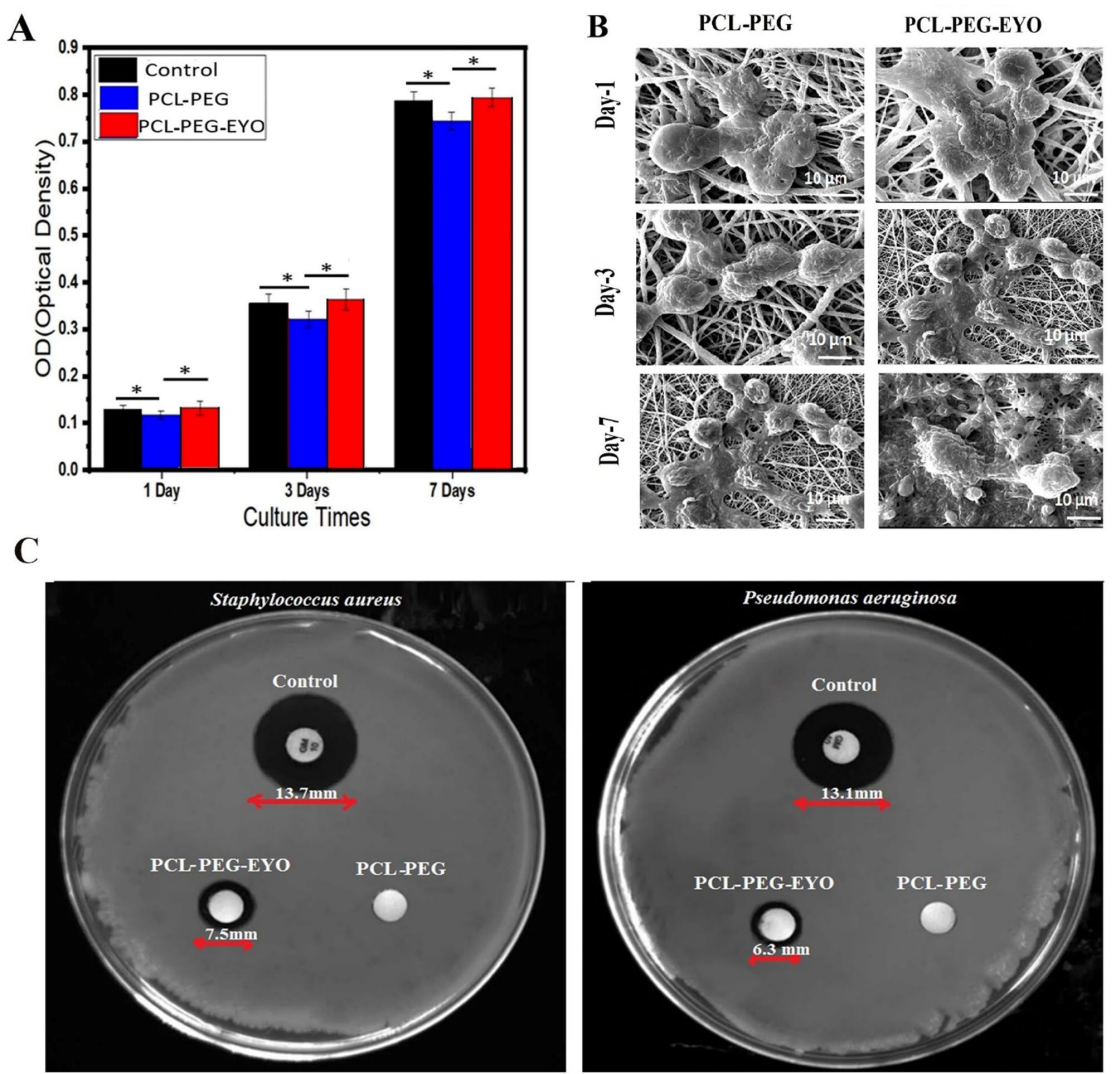
(**A**) MTT assay; (**B**) SEM micrograph of L929 cells cultured on PCL-PEG and PCL-PEG-EYO nanofibrous scaffold after 1, 3 and 7 day(s); *P-value < 0.05; (**C**) Antibacterial activity of (**A**) PCL-PEG-EYO web, (**B**) PCL-PEG web and (**C**) Control (*Gentamicin 10 mg*) against *Staphylococcus aureus* as a gram positive bacteria and *Pseudomonas aeruginosa* as a gram negative bacteria.

MTT assay was performed to evaluate PCL-PEG as well as PCL-PEG-EYO scaffolds' cytotoxicity against L929 fibroblast cells after 1, 3, and 7 days (Fig. 4A). In general, the optical density for both scaffolds rises with increasing time of culture exposure. The viability of cells onto the control group as well as PCL-PEG-EYO scaffold is higher than onto PCL-PEG scaffold (p < 0.05) in each time point. However, there is no significant different between the cell viability of PCL-PEG-EYO scaffold and control group (p > 0.05). So, the PCL-PEG-EYO scaffold could more effectively enhance proliferation of L929 cells on each time point compared to the PCL-PEG group.

The SEM images of scaffolds cultured with L929 cells are depicted in Fig. 4B. L929 cells could successfully attach on the surface of the PCL-PEG nanofibrous scaffold and continue to proliferate as the increasing of incubation time. This can be partly attributed to the high porosity and nanometer size of the scaffold’s fibers along with biocompatibility, bioavailability and wide safety profile of the PCL and PEG polymers. Moreover, presence of numerous essential nutrients in the ingredients of EYO, including lipids, cholesterol, biotin, B12, vitamin D, organic and inorganic elements, resulted in a better adhesion and penetration of cells inside the PCL-PEG-EYO scaffold, especially on day 7 post seeding^19,39^.

### Antibacterial activity of the nanofibers

Antibacterial activity of the scaffolds was performed by disc diffusion antibiogram test. The MIC of EYO against *Staphylococcus aureus* and *Pseudomonas aeruginosa* was about 10 μg/mL and 11 μg/mL, respectively. As depicted in Fig. 4C, no inhibitory zone was formed around PCL-PEG nanofiber which is representative of the lack of antibacterial of this scaffold against both gram positive and negative bacteria. Instead, the diameter of inhibition zone around PCL-EG-EYO scaffold was found to be 7.5 ± 0.4 mm and 6.3 ± 0.2 mm against gram positive and negative bacteria strains, respectively. This test was repeated 3 times for each group. The enhancement of antibacterial activity in PCL-EG-EYO scaffolds could be attributed to the presence of OH groups and phosphonic acid groups in the egg yolk oil^16^. Moreover, the higher antimicrobial activity of the oil-containing scaffold against gram-positive bacteria than that of gram-negative bacteria could be related to the difference of their cell wall structure. The permeable layer of gram-positive bacteria cell wall makes it more vulnerable against antiseptics than gram-negative bacteria with impermeable cell wall^40^.

### In vivo evaluation of the burn wounds healing

#### Macroscopic investigation

**Figure 5:**
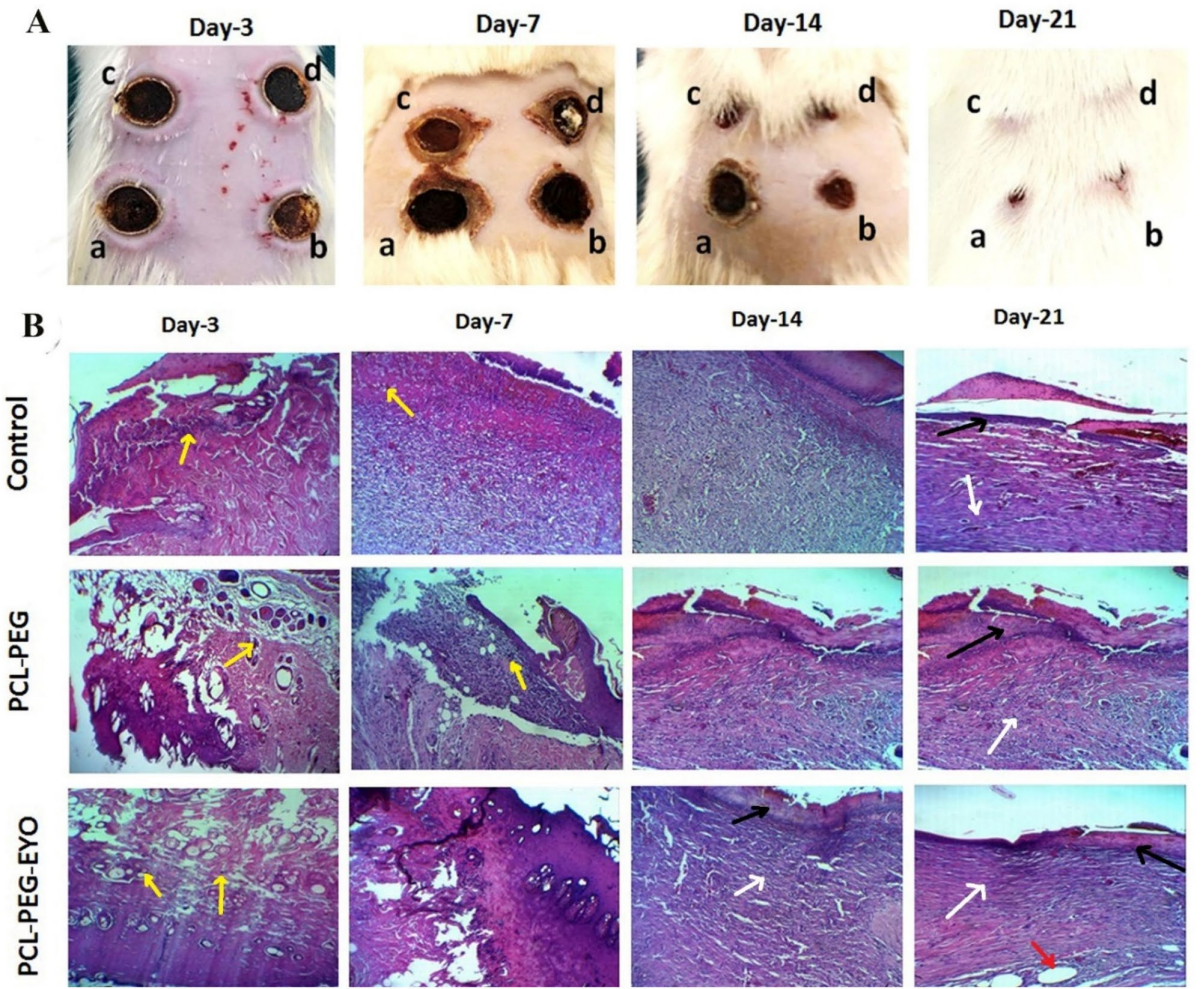
(**A**) Macroscopic investigation of wound healing process in different wounds treated with: (a) no scaffolds (control), (b) PCL-PEG nanofibrous scaffold, c and d) PCL-PEG-EYO nanofibrous scaffolds; through digital imaging at time points of 3, 7, 14 and 21 days post operating; (**B**) H&E histology images (100x) of wound tissue in control group, PCL-PEG and PCL-PEG-EYO nanofibrous scaffold. (Yellow arrows: PMNs; white arrows: collagen formation; black arrows: new epithelium; red arrows: skin appendages).

Evaluation of the rate of wound healing in PCL-PEG-EYO scaffold treated wounds compared to the PCL-PEG and control treated ones was performed by imaging the wounds on days 3, 7, 14 and 21 post induction of the 3rd degree burn wounds in rat models, utilizing a digital camera (Fig. 5A). The area of wounds at various treatment time-points has also been calculated by Digimizer software and reported in Table 1.

**Table 1:** The average wound area treated with different scaffolds at various time points.

Wounds treated with	Average wound area (mm^2^)			
	Day-3	Day-7	Day-14	Day-21
Control	71.02 ± 1.5	38.88 ± 1.6	27.69 ± 0.4	13.699 ± 0.7
PCL-PEG nanofibrous scaffold	59.5 ± 1.1	30.73 ± 0.62	17.52 ± 0.51	6.46 ± 0.6
PCL-PEG-EYO nanofibrous scaffold	51.8 ± 0.90	21.61 ± 0.53	9.33 ± 0.80	0.0051 ± 0.00

As mentioned earlier, the average diameter of the burn wound was about 10 mm after debridement. So, immediately after the wounds were created in all samples, the average debrided wound area was about 78.5 mm^2^. As depicted in Fig. 5A and Table 1, the apparent process of wound healing shows excellent contraction (significant reduction of the wound surface) of the wounds treated with the PCL-PEG-EYO nanofibrous scaffold. While wound closure in samples treated with PCL-PEG-EYO scaffolds was almost complete on day 21 post induction of burns, PCL-PEG scaffold and control treated wounds remained still open and demonstrated approximately similar wound area of 6.46 ± 0.6 mm and 13.699 ± 0.7 mm^2^, respectively. This represents the superiority of PCL-PEG-EYO nanofibrous scaffolds in acceleration of full-thickness burns healing.

#### Microscopic (pathological) investigations

**Figure 6:**
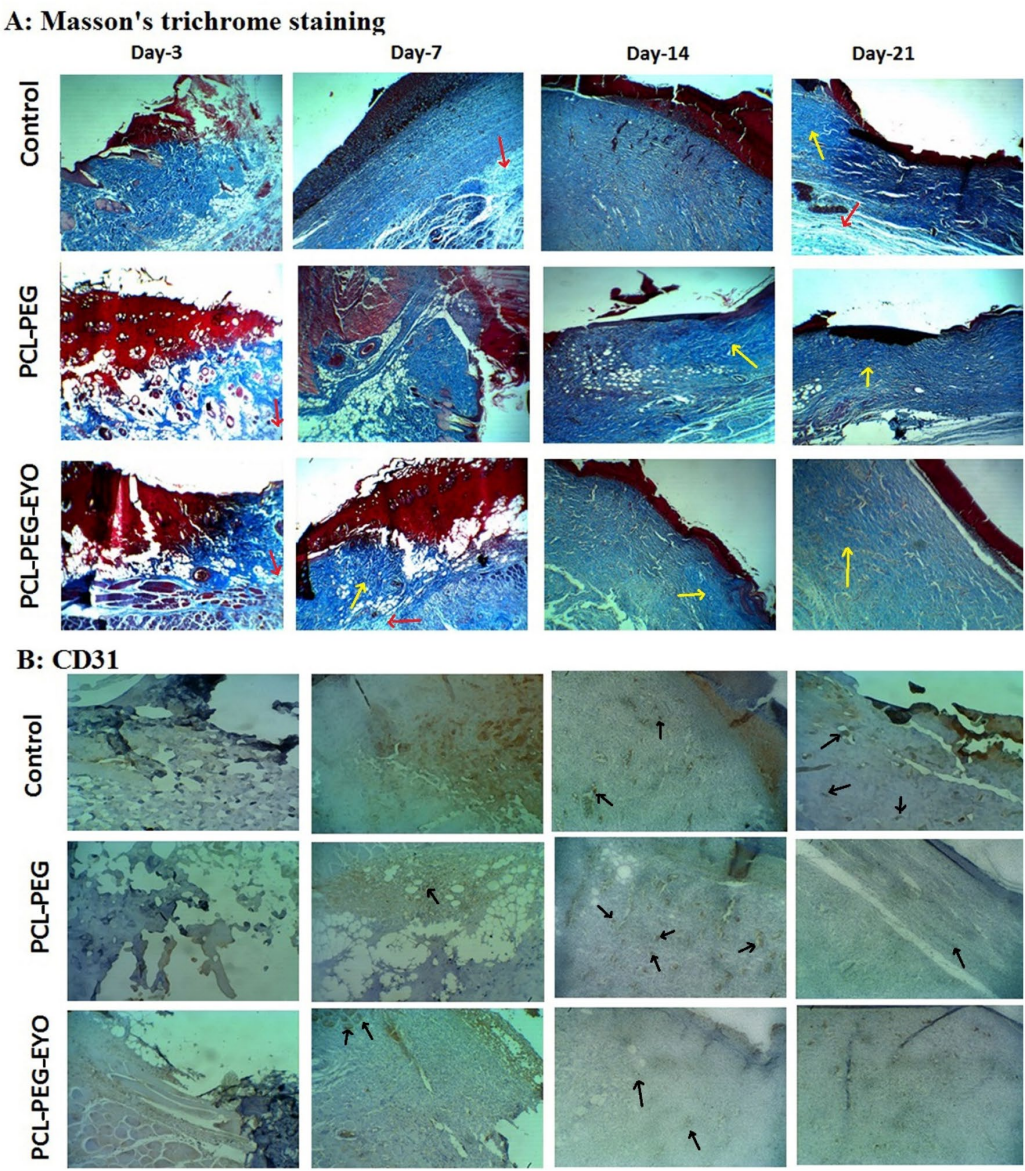
(**A**) Masson's trichrome staining (100x) and (**B**) CD31 marker for investigating of collagen synthesis (Red arrows: fine collagen fibrils; Yellow arrows: collagen fibers) and angiogenesis (Black arrows) histology of burn wound tissue in control group, PCL-PEG and PCL-PEG-EYO nanofibrous scaffold at different time-point post treating.

Figures 5B and 6 show histological images of wound sites under H&E staining and Masson's trichrome followed with CD31 marker staining, respectively. The former is the most popular methods for tracking healing process (Fig. 5B) such as re-epithelialization and the latter is used for evaluation of the collagen synthesis followed with angiogenesis (Fig. 6). In order to follow a detailed trends in healing process according to Table S1^28^, the scored values of different effective criteria in burn wound healing are reported in Table 2.

**Table 2:** Results of scored criteria based on Table S1 according to the results of Fig. 5B and Fig. 6.

Burn treated with	Day post treating	Score of criteria				
		Epithelialization	PMNL	Fibroblasts	New vessels	Collagen
Control	Day-3	0	1	0	0	0
	Day-7	0	1	1	1	0
	Day-14	0	0	2	2	1
	Day-21	2	0	2	2	1
pcl-PEG nanofibrous scaffold	Day-3	0	1	0	0	0
	Day-7	0	0 to 1	1–2	1	1
	Day-14	1	0	2	1	1
	Day-21	2 to 3	0	2	2	2
PCL-PEG-EYO nanofibrous scaffold	Day-3	0	2	0	0	0
	Day-7	1	0	2	1	2
	Day-14	2 to 3	0	2	2	2
	Day-21	3	0	3	1	3

According to Table S1, the re-epithelialization process is scored between 0 to 4, with zero referred to the state of “no epithelialization” or “no signs of burn wound healing” and 4 referred to “full epithelialization”. Hence, the higher the re-epithelialization score, the better the outcome of the therapy would be. Considering inflammatory responses in the burn wound healing process, an exaggerated response is expected up to the end of the inflammatory phase (the first 3–5 days from injuries) which is mostly due to the enhancement of the infiltration and proliferation of neutrophils and polymorphonuclear neutrophil cells (PMNCs) in the wounded area. Beginning from day 5, inflammatory responses will begin to eventually subside (owing to a reduction in the number of PMNCs and their replacement with lymphocytes and macrophages) up to the end of the day 14 and will completely resolved by day 21 post injury. Detection of any PMNCs in the wounded area from day 10, is representative of formation of “abscess” in the injured area and is a negative score for wound healing. Similarly, detection of mononuclear cells in burn wounds from day 14–15, is the sign of an active wound and again, is a negative score for healing. Fibroblasts are among the main collagen-producing cells in the dermis which begin to proliferate from day 3 post injury up to the end of day 14, in response to the secreted growth factors by PMNCs.

The ultimate outcome of fibroblasts proliferation is epithelialization and closure of the wound bed through contraction of the fibrocystic-fibroblastic proliferative compartment. Hence, higher scores for the number of fibroblasts at the wounded area are informative of a better wound healing process.

Angiogenesis or the process of the formation of new vessels from preexisting ones, is an event taking place along with proliferation of the fibroblasts during burn wound healing process. In this context, angiogenesis continues up to the end of the third week when wound contractions begin. Consequently, an intensive angiogenic response up to the end of the third week is beneficial for healing while from there on, continuation of angiogenesis is demonstrative of an impairment wound healing process and presence of active wounds. Finally, collagen synthesis and deposition takes place along with maturation of fibroblasts and their transformation into fibrocytes. Therefore, higher score for collagen synthesis and deposition is also informative of a better wound healing process^28^.

Table 2 represents data obtained from microscopic evaluation of sections prepared from burn injuries treated by no scaffolds (control), PCL-PEG and PCL-PEG-EYO nanofibrous scaffolds. Considering molecular events up to the end of day 3 post burn injuries, score 2 and score 1 inflammatory responses were observed with PCL-PEG-EYO and PCL-PEG treated groups respectively, while no signs of re-epithelialization, existence of fibroblasts, angiogenesis and collagen synthesis were presented in either of the groups. Hence, whole groups were almost the same up to the end of the day 3 post injuries.

On day 7 post injury, while inflammatory cells were still presented in control group, a significantly attenuated inflammatory response was observed in PCL-PEG and PCL-PEG-EYO treated groups. Moreover, although the number of fibroblasts in control group was low, PCL-PEG and PCL-PEG-EYO treated wounds demonstrated a statistically significant rise in number of fibroblasts in the wounded area. Noteworthy, the extend of angiogenesis and collagen deposition were significantly more in PCL-PEG-EYO group compared to the other studies groups which demonstrates superior advantageous effects of PCL-PEG-EYO compared to the others.

Considering molecular events in control group on day 14, despite of a subsidiary in inflammatory responses, no meaningful progress in re-epithelialization process and fibroblast migration from wound edges were recorded. Moreover, fibroblast cells were moderately increased and angiogenesis was scarcely increased. Similarly, collagen deposition was negligible and weekly observed. Regarding PCL-PEG dressing, cell migration from edge of the wounds was initiated, collagen deposition and angiogenesis were significantly low in intensity compared to the PCL-PEG-EYO treated wound. Interestingly, PCL-PEG-EYO treated wounds demonstrated a clear collagen synthesis process and a homogenous deposition pattern at the healing site.

Evaluation of wounds of control group on day 21 demonstrated a significantly enhanced re-epithelialization process together with active angiogenesis and collagen synthesis processes. Consequently, wounds in control group were considered to be new and healing process was poor and did not progressed well. Regarding PCL-PEG treated group, re-epithelialization process was progressed well and endothelial cells could form a narrow bridge between edge of the wounds for induction of contracture and closure. Nevertheless, collagen synthesis and deposition were moderately happened and were significantly lower than those observed with PCL-PEG-EYO group. Considering PCL-PEG-EYO treated wounds, epithelialization process was almost completed and homogenously synthesized collagen fibers were transformed to collagen bundles in the wounded area. Moreover, as depicted in Fig. 5B, formation of skin appendages at the burned site on day 21 post treatment is representative of the high quality of the healing process in wounds treated with PCL-PEG-EYO dressing.

## Conclusion

In summary, we reported successful fabrication of PCL-PEG-EYO nanofibrous scaffold through electrospinning technique. The molar ratio of PCL to PEG was set to 1:1 in DCM/DMF (9:1 v/v). The optimum condition for electrospinning process of the scaffold was found to be 20 kV and the distance from tip to collector of 15 cm. The PCL-PEG-EYO demonstrated an acceptable hydrophilicity with high cell viability and cell attachment up to 7 days after cell culturing. Moreover, such scaffold showed antibacterial activity against both gram positive and negative bacteria due to the presence of EYO. Finally, in vivo evaluation revealed improvement of the rate of wound closure, as well as re-epithelialization and collagen deposition after 21-day treatment period using PCL-PEG-EYO nanofibrous scaffold. Overall, the PCL-PEG-EYO nanofibrous scaffolds demonstrated a great potential in management of full thickness burn wounds in vivo.

## Supplementary Information

Supplementary Information.

## Data Availability

All data generated or analyzed during this study are included in this published article (and its Supplementary Information files).
