# Supplementary material for: Preclinical evaluation of the polycaprolactone-polyethylene glycol electrospun nanofibers containing egg-yolk oil for acceleration of full thickness burns healing
Source: Sci Rep. 2023 Jan 17;13:919. doi: 10.1038/s41598-023-28065-6 (PMC9845205; doi:10.1038/s41598-023-28065-6)
Supplement: Supplementary file 1 — Supplementary Information. [file 41598_2023_28065_MOESM1_ESM.docx]

**Supplementary information**

Supplementary of manuscript entitled:

**Preclinical Evaluation of the Polycaprolactone-Polyethylene Glycol Electrospun Nanofibers Containing Egg-Yolk Oil for Acceleration of Full Thickness Burns Healing**

**Fig. S1.**


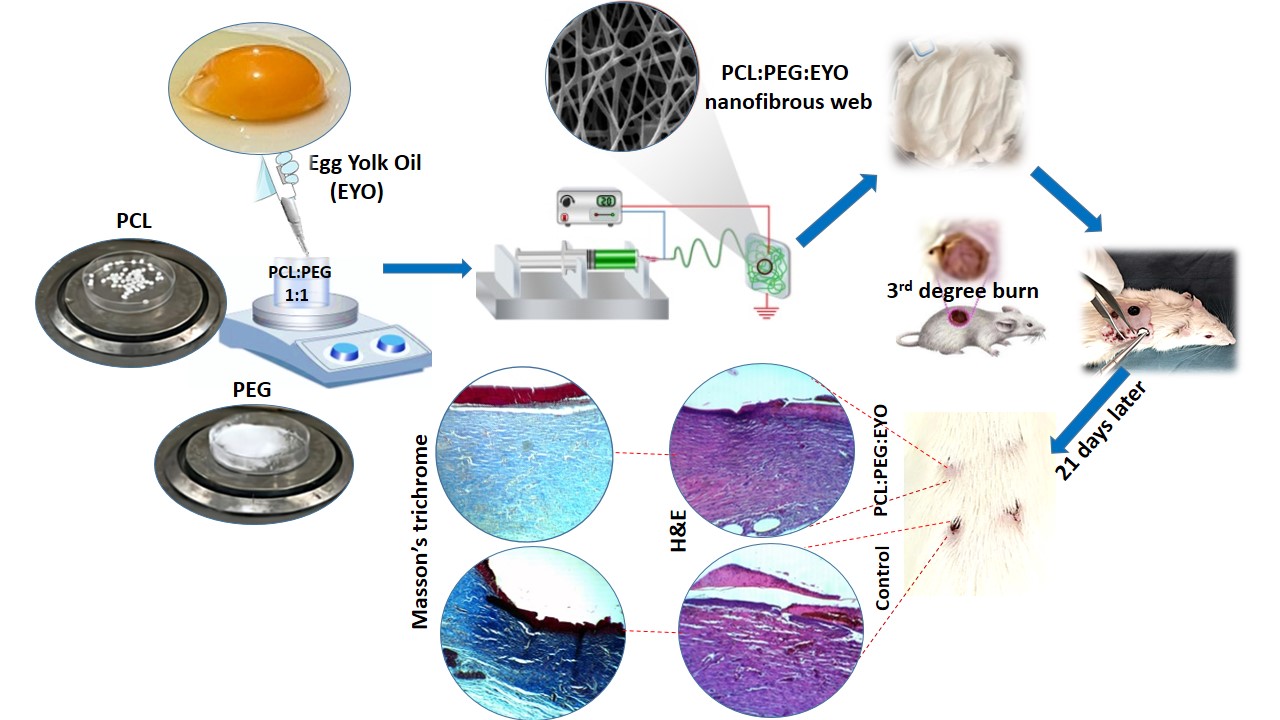
**Fig.S1.** Schematic of scaffold fabrication and its application on full-thickness burn on rat model dorsum skin and the efficiency of healing using digital imaging and pathological studies.

Table S1. The scoring of healing criteria based on reference [28].

| Epithelialization | PMNL | Fibroblasts | New vessels | Collagen |
| --- | --- | --- | --- | --- |
| Thickness of cut edges =0 | Absent=0 | Absent=0 | Absent=0 | Absent=0 |
| Migration of cells (< 50%) =1 | Mild  (Less than 25%) =1 | Mild (surrounding tissue)=1 | Mild (surrounding tissue) =1 | Mild (surrounding tissue)=1 |
| Migration of cells (≥ 50%) =2 | Mild  (25-50%) =2 | Mild granulation tissue =2 | Mild granulation tissue =2 | Mild granulation tissue =2 |
| bridging the excision =3 | Moderate  (50-75%) =3 | Moderate granulation tissue =3 | Moderate granulation tissue =3 | Moderate granulation tissue =3 |
| Keratinization =4 | Marked  (over than 75%) =4 | Marked granulation tissue =4 | Marked granulation tissue =4 | Marked granulation tissue =4 |

**Fig. S2.**

**
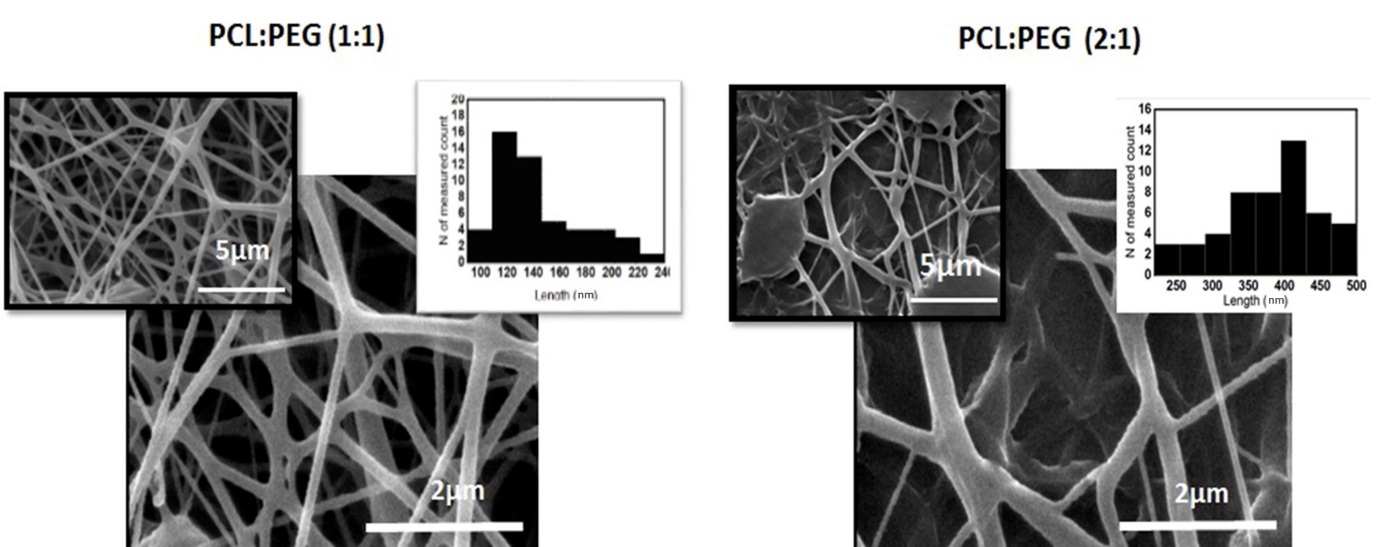
**

**Fig. S2.** SEM images of PCL-PEG nanofibers with different PCL: PEG mass ratios of 1:1 and 2:1 under applied voltage of 20 kV and distance to the collector of 15 cm.
